# Critical Reflection on the Use in Canada of the Canadian Model of Occupational Performance and Engagement

**DOI:** 10.1155/oti/1400316

**Published:** 2026-06-17

**Authors:** Annie Rochette

**Affiliations:** ^1^ School of Rehabilitation, Faculty of Medicine, University of Montreal, Montreal, Quebec, Canada, umontreal.ca; ^2^ Centre for Interdisciplinary Research in Rehabilitation of Greater Montreal (CRIR), Montreal, Quebec, Canada

**Keywords:** CMOP-E, identity, relations, spirituality, virtual environment

## Abstract

**Background:**

Through the teaching of the Canadian Model of Occupational Performance and Engagement (CMOP‐E), a number of repeated challenges were witnessed. The purpose was to critically reflect, through dialogue, on potential additions to align with societal evolution.

**Process:**

The application of the CMOP‐E was discussed with occupational therapy students for over more than 20 years of teaching at both the undergraduate level and the professional master′s level in occupational therapy. Three key issues repeatedly emerged and triggered an in‐depth reflection. The content of this reflection was discussed with a variety of senior academics holding expertise in the CMOP‐E.

**Key Issues:**

Arguments supporting the idea that it would be of value to make explicit the concept of “identity” within the spirituality dimension are proposed. The environment should be considered at three levels: micro, meso, and macro levels, and a reflection is presented on the space that should be allowed for “virtual” as another category of environment. Lastly, “relationships” should be explicitly considered as a domain of occupation and not as an affective state or the social environment.

**Implications:**

Making these three “additions” to the description of the model will help to clarify how the CMOP‐E can be used in contemporary practice.

**Summary**

• An in‐depth reflection on how to extend the CMOP‐E to contemporary practice is warranted.
• The term “identity” could be used as an adjunct to the spirituality dimension and the virtual environment, although being omnipresent should not be considered as a distinct element.
• Relations are occupations of their own and should not be confused with an affective state or the social environment.

## 1. Introduction

The Canadian Model of Occupational Performance and Engagement (CMOP‐E) [1] is one of the most widely used models in Canada. Indeed, in a descriptive study looking at the enactment of competencies among occupational therapists holding an entry‐to‐practice master′s degree in Quebec, the CMOP‐E was reported as the top model used by 67.8% (215/317) of respondents [2]. As such, despite the emergence of new models—such as the Canadian Model of Occupational Participation (CanMOP) ([3])—and considering the time lag to implementation, the CMOP‐E probably remains widely used in practice and is still part of all Canadian occupational therapy university curricula. However, the CMOP‐E is not static. It has constantly changed in the past [4], mirroring societal evolution, as has the occupational therapy profession. The first version of the CMOP‐E, included in occupational therapy guidelines, was called the Model of Occupational Performance [5]. This model was illustrated as three circles with the individual at the center (including spiritual, physical, mental, and sociocultural dimensions), surrounded by the three occupational domains (self‐care, productivity, and leisure), and with the outer circle comprising the environment (social, physical, and cultural). Its main differences from the current iteration were the absence of the construct of engagement and the absence of the institutional elements in the environment. Moreover, although the original model included four dimensions of the individual (namely, spiritual, physical, mental, and sociocultural), these differ from the current dimensions, which are spirituality, physical, cognitive, and affective. These occupational therapy guidelines, anchored into the Model of Occupational Performance, were further updated in 1991 and 1993 without major changes to the model. However, in 1997—the same year as the first publication of the *Profile of Occupational Therapy Practice in Canada*, the Canadian competency framework of that time for the profession—the Canadian Model of Occupation Performance (CMOP) was introduced [6]. In the CMOP, the inner circle becomes a triangle with spirituality at the center (at the core), and with the physical, cognitive, and affective dimensions each located at an angle of the triangle. The CMOP is aimed at emphasizing the construct of occupational performance as a result of the interactions between the person, their occupations, and their environment.

The changes made to the CMOP‐E as following societal evolution was mentioned upfront. It might be worth to recall that in the 1970s and 1980s, groups came together to advocate for the rights of the disabled persons; the Declaration for the Rights of Disabled Persons was first issued in 1975 by the United Nations. These groups advocated for considering the environment as a determinant of disability, which gave rise years later to interdisciplinary models. Indeed, the first version of the actual Human Development Model—Disability Creation Process [7] was first introduced in 1995, and the well‐known International Classification of Functioning, Disability, and Health (ICF) was first introduced in 2001. Those two interdisciplinary models use the construct of participation that can be considered as a proxy for doing occupations. Interestingly, in the transition of the CMOP becoming the CMOP‐E (E for engagement), it went through a very short period, some of us might recall, where it was called the CMOP‐P (P for participation) to rapidly endorse the term engagement as a construct. The construct of engagement was aimed at capturing the broadest perspective on occupation, beyond performance [1]. With the constructs of participation and engagement, it became even clearer that there is no normal way (i.e., common pattern, following a set of rules or standards, according to Antidote) across individuals to accomplish a given occupation. Instead, this accomplishment can be labeled as optimal for a given individual only when there is a perfect congruence between the way one aspires to accomplish it and the actual way it is (or can be) accomplished in reality [8]. As such, it might be argued that the qualifiers of performance and engagement are not even required to truly, fully, and clearly describe the absence or the amplitude of a gap between an ideal and actual way of doing occupations. On a side note, this essential comparison between an ideal and an actual way of doing (being, belonging and becoming) does not apply to the three performance components/dimensions (physical, cognitive, and affective) of the person where norms as to what is expected across individuals exist.

Society is still evolving, and the 2000s were characterized by growing societal interests (or concerns) relating to individuals′ identity [9], environmental concerns [10], and the importance of relationships [11], especially with the rapid emergence of social media embedded in a virtual environment along with social deprivation and isolation concomitantly to the COVID‐19 pandemic. Meanwhile, through teaching the CMOP‐E to learners who have to apply it in practice, a number of repeated challenges mirroring those societal interests/concerns were witnessed.

## 2. Purpose

The purpose here was thus to reflect on key issues faced by learners when using the CMOP‐E in practice. The issues repeatedly found to be difficult for learners are to: (1) apply and use the spirituality dimension in practice; (2) embrace the scope of environmental characteristics important to consider in a given situation, including the space allowed to the virtual environment; and (3) consider relationships as an occupation.

### 2.1. Systematic Approach to the Construction of Arguments

This is not a research paper but rather an in‐depth critical reflection [12]. As such, ethic′s approval was not required. To contextualize, the author is an academic leading research in occupational therapy and rehabilitation and has been discussing the application of the CMOP‐E with students in occupational therapy for more than 20 years of teaching at the undergraduate level and the professional master′s level. How this experience may have shaped her receptivity for a newer model remains hard to document. The publication of a new book in late 2022, including a new model, the CanMOP [3], which was confusedly presented as the “new Canadian guidelines” at that time, contributed to triggering the need for this in‐depth critical reflection using a dialogical process of communication with learners, academic colleagues, and CMOP‐E experts [12]. Indeed, although ideas/themes highlighted in the book (e.g., importance of the therapeutic relationship, using phenomenological or narrative approaches to better understand what our clientele really needs and wants from their individual and collective perspective or the importance of considering the micro, meso, and macro environment into the occupational therapy analysis) make sense, its content did raise a number of areas of concerns. These concerns were shared with the two coeditors of the book [3] and discussed further informally with Canadian academic colleagues from 11 different universities between January and April 2023. These colleagues were contacted first by email and the majority provided their input through a conversation using videoconferencing. All had actual or previous involvement in teaching models and were identified by words of mouth. These inputs were collected informally through individual conversations, and on‐the‐fly syntheses were written down in a single Word file taking the form a logbook (i.e., it was not part of a research study). As it is not the purpose of this paper to comment further on this, there were significant concerns with their approach suggesting that all literature, all models, and all developments in our profession over the last 20–30 years ought to be discarded in favor of a completely new slate, which contributed highly to deepen the current reflection on how older models, such as the CMOP‐E, could be improved to follow societal evolution. Three key issues, one per its main components, were repeatedly emerging throughout this in‐depth reflection. The specific content of this reflection was discussed through four meetings in 2023–2024 with a variety of senior academics (i.e., already retired or semiretired) holding expertise in the CMOP‐E (i.e., holding many peer‐reviewed publications on the CMOP‐E).

## 3. Key Issues

### 3.1. Spirituality and Identity

The spirituality dimension has been perceived as a difficult component to apply in practice for a long time [13]. Nowadays, this dimension seems to be too often neglected, especially by learners. It is not rare that learners simply overlook this dimension when reporting on cases in an academic environment or apply it in a reductionist manner, as a synonym of religion. Yet, originally, there were discussions about the choice of the word spiritual, which further became spirituality, for which the term “personal essence” was proposed but not retained. When looking at the definition of the word spirituality, this confusion with religious beliefs makes sense (according to Antidote, spirituality is defined as *the state or quality of being spiritual*; and spiritual is defined as *of or referring to the human spirit or soul*, *as opposed to the physical body or mind*, *of or referring to religion or religious values*, *beliefs*, *etc.*). The 2000 Muriel Driver Memorial Lecture was on the topic of spirituality, where McColl [14] provided deep thoughts and analysis around this concept and its implication for practice. Yet, more than 20 years later, its application in practice remains a challenge.

Considering the evolution of Canadian society and the omnipresence of the Justice, Equity, Diversity, and Inclusion concepts at all governmental levels as well as the evolution of our profession mirroring society, the addition of the word “identity” as a personal essence to the spirituality dimension could make this dimension more explicit about what it truly means, in essence and how practitioners need to apply it in practice. Spirituality is at the core of the full model and is considered here as an intrinsic component of the person, although there are debates about spirituality also being a component of the environment (extrinsic components) or of the doing of occupations [4]. A proposition to re‐examine spirituality as occupational identity was proposed in the early 2000 [15]. Indeed, the word identity is defined according to Antidote as *the qualities*, *beliefs*, *attitudes*, *etc. that make one person or group distinct from another*. In practice, it is essential to get a deep understanding of who the person (or the population) really is, what they value the most, what is most meaningful/significant for them, and how they describe, perceive, and see themselves, as this is also key information to apply the JEDI principles. On a side note, other models also have this construct included. For example, this is referred to identity factors embedded into personal factors in the Model of Human Development—Disability Creation Process [7], and into personal factors in the ICF [16]. The constructs of interests, values, and roles of the MOHO [17] also provide valuable information about a person′s true essence. As emphasized by Egan and Delaat [18] more than 30 years ago, the most important would be to allow spiritual expression that can be realized through getting to know our clients well, finding out how clients connect, and being sensitive to their own feelings, values, and beliefs.

Indeed, acknowledging that everyone is unique reinforces the importance for occupational therapists to reflect on their own preferred posture regarding pluralism. Interestingly and not related to the CMOP‐E per se, White [19] proposed a 3‐D lens as a simple analysis grid to understand potential tensions in interculturalism contexts. The 3‐D stands for the recognition of Diversity, the fight against Discrimination, and reconciliation through Dialogue; where a given individual usually would show a stronger tendency towards one of these 3‐D, with a complementary second favored stream, and the third one perceived as problematic or irritating. As an example of application, when looking at the wording used in the Domain C (Culture, Equity, and Justice) of the most recent document of Canadian occupational therapists′ competencies [20], the antidiscrimination lens is obvious. The posture a clinician would adopt primarily in a given context would necessarily influence their choice of action to address the potential gap between self and client in relation to pluralism, which would be operationalized as spirituality/identity in this version of the CMOP‐E. Thus, the addition of the construct of identity to the spirituality construct could better equip occupational therapists to practice ethically with culturally diverse clients.

### 3.2. Environment as Micro, Meso, and Macro and the Space Allowed to the “Virtual”

The second issue for learners reflected on is their difficulty to embrace the scope of environmental characteristics important to consider in a given situation, including the space allowed to the virtual environment. One challenge with the environment lies within its large scope and being conceptualized in the CMOP‐E as four elements: physical, social, institutional, and cultural. Being so large and having the potential to take so many forms, learners tend to consider it insufficiently or present characteristics/factors at only one level, for example, focusing on home accessibility or living situation, that is, living or not with family members. To help resolve this challenge, the three levels of micro, meso, and macro, using the definitions of Bronfenbrenner′s bioecological theory [21] could be added to the environment component. These three levels are explicitly included into the environmental factors of the Model of Human Development—Disability Creation Process [7] and in the recent CanMOP [3]. Concretely, the environment could then be considered according to two axes: the type of environment using the actual four elements of physical, social, institutional, and cultural as well as the three levels of micro, meso, and macro. This is quite simple to add and may help to consider its key characteristics potentially interacting with the person and their occupations, through different lenses, on different layers.

Furthermore, in pursuing the reflection on how society evolves and how this might contribute to the CMOP‐E evolution, a reflection as to whether the virtual environment, being now omnipresent, should be considered as a distinct element of its own seemed to be warranted. However, what is a virtual environment and what makes it distinct from real environment? As with the majority of constructs, the environment cannot be described in a dichotomous manner, simply as being real or virtual but should be described on a continuum [22], with at one end, the real environment, and at the other end, the virtual environment and with a number of variations in‐between integrating both, such as augmented reality, pure‐mixed reality, or augmented virtuality [23].

Because a virtual environment (or a “nonreal environment”) requires some form of physical devices to exist, that although it becomes omnipresent in contemporary society and that many occupations can either be accomplished along the continuum of real to virtual environment, there is no need to add a specific element relating to it in the CMOP‐E. The main argument is that the required devices to characterize an environment as “nonreal” are part of some form of technologies, which are embedded into the physical elements by nature, and thus, the virtual environment would already be positioned within the physical environment. Devil′s advocate could argue that nowadays, it is even possible to interact with a virtual environment [24], does that make this element social instead of physical? May be. A similar argument could be made with domestic animals. Are they part of the physical or social elements of the environment? The same reasoning could be applied as the one applied to the occupation construct in the CMOP‐E: it is the person with whom the CMOP‐E is used that is better positioned to decide into which category a given occupation belongs (e.g., does someone brush teeth for self‐care or for productivity if they are a dental hygienist) or in this case, to decide into which category a given element of the environment belongs (e.g., communication robot as a physical or social element of the environment).

### 3.3. Relationships as an Occupation

The third and last key issue is about the difficulty for learners to consider relationships (understood here as social interactions) as an occupation. Relations are a meaningful occupation for most people if not all [25]. However, a confusion remains for many learners, practitioners, and even academics as to whether relations are an occupation and even if these can be considered as an occupation in itself without necessarily being a component of an activity. The fourth edition of the Occupational Therapy Practice Framework [26] does not really consider social interactions as an occupation of its own but does include social participation as one of its domain defined as engaging in activities involving social interactions with others. Indeed, being in a relationship as an occupation is likely to be confounded with the social elements or even the affective dimension of the CMOP‐E. In models stemming from classifications such as MHD‐DCP [7] or the ICF, that is, in models where participation/occupation is described through categories, relations represent a category of participation in itself and include a number of different subcategories such as sexual relationships. As with other occupations, there is not only one expected way of being in relationships, and thus, the quality and the quantity (frequency) of the relation can be used as descriptors. Relations (similarly to other occupations) can be considered using a “timeline” prism such as the process of creating, maintaining, and ceasing a relationship. It can also be considered and described regarding to with whom the relationship is taken place, for example, spouse, family members, intimate relationship, friends, professional relationships, or with strangers.

The person with whom one is in relation with represents the social environment. However, one must be cautious as to not solely name the link (e.g., friend, parent, and spouse) that person has as this is not enough to fully capture how this social environment is likely to influence (or be influenced by) the relationship as an occupation. Characteristics of this person such as their attitude, behaviors, and availability may inform better. In a similar way, the affective dimension (e.g., how one feels) is not a relationship in itself but will likely be influenced by the quality of the relationships (and vice‐versa).

A gap in ideal versus actual way relations are experienced is most of the time present when a person is referred for occupational therapy [25]. Yet, for learners, relations are rarely considered or assessed as an occupation, and therefore even less a target of intervention; this is an important gap in practice that needs to be addressed. This gap in practice is well documented specifically for sexual relations [27]. The difficulty observed with learners to consider relationships when using the actual three domains of the CMOP‐E, self‐care, productivity, and leisure, may contribute to this gap as relationships do not fit intuitively in either of these three domains.

One argument heard against adding a fourth domain of occupation specifically targeting relations was that relations are most of the time part of other occupations, and this is indeed the way they are defined in the fourth edition of the Occupational Therapy Practice Framework [26] as engaging in activities involving social interactions with others. Yet, this is also similar for communication and mobility [28], but these latter are considered occupations in themselves. An argument against this would be that relations may indeed be a prerequisite for other occupations, but other occupations are often accomplished and valued because they provide an opportunity for relationships [25]. An example that was brought up in class by a learner was the case of a teenage boy who wanted to go back to school after a head trauma; he did not value schooling activities as much per se but school represented the context where he could interact with his friends and therefore, what he wanted the most was not as much as getting back to school but rather to interact with his friends. Another example is people who interact only when they go shopping with the introduction of a “slow cashier” [29], specifically to allow for relationships in the context of shopping and decrease isolation. Relations are not an intrinsic characteristic of an individual (included into the person component) although they are likely to influence how ones feel (and vice‐versa) nor are they an extrinsic characteristic, and as such, relations are components of the doing of occupations, further likely to contribute to their being, belonging, and becoming [30]. They can be described on a continuum from a single interaction to long‐lasting intimate relationships. Relations are commonly happening with another human being, although relations to domestic animals are also common [31], and we may witness more and more relations to robot or AI‐assistant in the future [32].

## 4. Implications

In summary, an extended version of the CMOP‐E is proposed with minor additions such as the construct of identity to complement the spirituality dimension, the consideration of the micro, meso, and macro levels to describe the environment, and the explicit recognition of relations as a domain of occupation. These simple additions should contribute to a better application of the CMOP‐E in practice, especially for learners who persistently faced challenges around those three issues. This reflection was limited to a Canadian context and more specifically within learners from the Province of Quebec. As such, the relevance of this content for the global community using the CMOP‐E elsewhere in the world would require further reflections.

## Funding

No funding was received for this manuscript.

## Conflicts of Interest

The author declares no conflicts of interest.

## Data Availability

Data sharing is not applicable to this article as no datasets were generated or analyzed for writing this manuscript.
